# Cancer Immunotherapy and the Immune Response in Hodgkin Lymphoma

**DOI:** 10.3389/fonc.2018.00193

**Published:** 2018-06-04

**Authors:** Christoph Renner, Frank Stenner

**Affiliations:** ^1^Department of Biomedicine, University Basel, Basel, Switzerland; ^2^Department of Oncology, University Hospital Basel, Basel, Switzerland

**Keywords:** Hodgkin lymphoma, monoclonal antibodies, bispecific antibodies, immunotoxins, check-point blockade inhibitors, chimeric antigen receptors

## Abstract

Patients with classical Hodgkin lymphoma (cHL) have an impaired cellular immune response as indicated by an anergic reaction against standard recall antigens and a diminished rejection reaction of allogeneic skin transplant. This clinical observation can be linked to the histopathological feature of cHL since the typical pattern of a cHL manifestation is characterized by sparse large CD30^+^ tumor-infiltrating Hodgkin–Reed–Sternberg (HRS) cells that are surrounded by a dense inflammatory immune microenvironment with mixed cellularity. Despite this extensive polymorphous inflammatory infiltrate, there is only a poor antitumor immune response seen to the neoplastic HRS cells. This is primarily mediated by a high expression of PD-L1 and PD-L2 ligands on the HRS cell surface which in turn antagonizes the activity of programmed death-1 (PD-1) antigen-positive T cells. PD-L1/L2 overexpression is caused by gene amplification at the 9p24.1 locus and/or latent Epstein–Barr virus infection present in around 40% of cHL cases. The blockade of the PD-L1/L2–PD-1 pathway by monoclonal antibodies can restore local T cell activity and leads to impressive tumor responses, some of which are long lasting and eventually curative. Another feature of HRS cells is the high CD30 antigen expression. Monoclonal antibody technology allowed for the successful development of CD30-specific immunotoxins, bispecific antibodies, and reprogrammed autologous T cells with the first one already approved for the treatment of high risk or relapsed cHL. Altogether, the discovery of the described pathomechanism of immune suppression and the identification of preferential target antigens has rendered cHL to be a prime subject for the successful development of new immunotherapeutic approaches.

## Biology of Classical Hodgkin Lymphoma (cHL)

Hodgkin lymphoma (HL) is a rare lymphoma entity with 3–5 new cases/100,000 inhabitants. The histopathological picture is unique as usually a few (1% or less of all cells) malignant cells called Hodgkin–Reed–Sternberg (HRS) cells are surrounded by a strong inflammatory cellular component (1, 2). The survival of HRS is highly dependent on the interaction with surrounding inflammatory cells since they do not survive as single cells when taken into cell culture (3, 4). The composition of the inflammatory cell compartment can vary substantially and defines the four histopathological subtypes of cHL (1). The origin of HRS cells was debated controversially and it is believed nowadays that they originate from germinal center B cells although they lack most B cell markers (5, 6). The recognition of HRS cells by the immune system such as cytotoxic T cells or T helper (Th) cells is dampened as they frequently downmodulate MHC-I and MHC-II molecule expression (7). In addition, they produce cytokines such as CCL5 and MIF that attract macrophages and mast cells. HRS cells stimulate M2 macrophages to produce MIF which in turn stimulates HRS cells through binding to the constitutively expressed CD74 antigen to even increase MIF production (4). In addition, HRS cells secrete CCL17 and CCL22 that recruit immunosuppressive Tregs into the cHL microenvironment which support the evasion of an immune attack (8). These cellular and soluble factors contribute to the special immune-evasive phenotype of cHL that is orchestrated to a large extent by HRS cells and could explain why attacking HRS cells might restore immunological control.

## Immune Deficiency and Immune Evasion in HL

It has been known for a long period of time that viral and fungal infections are increased in patients with cHL (9). In parallel, cHL patients have a decreased delayed type hypersensitivity reaction and were shown to be anergic against standard recall antigens including a diminished rejection reaction for allogeneic skin transplants (10, 11). A vast body of literature has accumulated on different aspects of a depressed T lymphocyte response *in vitro* to phytohemagglutinin, concanavalin A (Con A), and pokeweed mitogen (12, 13). These functional abnormalities correlate with the severity of the disease and are of prognostic relevance. The T-cell deficiency in cHL was assumed to be caused by qualitative defects as lymphocyte counts in cHL patients did not differ significantly from healthy controls. The qualitative defects were detected by a decreased proliferative response on stimulation with standard mitogens and the secretion of significantly lower amounts of interleukin-2 (IL-2) (14–17). The reduced IL-2 levels could not be explained by reduced IL-2 receptor expression in T cells from cHL patients. The observation of a decreased activity of the enzymes adenosine deaminase and 5′ nucleotidase in Hodgkin T cells, both essential for adequate T-cell proliferation, supported the hypothesis that cHL patients have an intrinsic defect for enzymes with relevance for T-cell function (18, 19). The same enzyme defect was found in other Epstein–Barr virus (EBV)-positive tumors, and it was speculated that the EBV infection was the common causative link for the observed immunodeficiency. Already at that time, researchers believed that HRS cells would express certain molecules, either as soluble factors or membrane bound that hampers the efficacy of T cell-mediated antitumor immune responses. For example, HRS cells express the immunoregulatory glycan-binding protein, galectin-1, which supports a Th2 regulatory immunosuppressive tumor microenvironment (20). Nowadays, we believe that the detection of variable amounts of programmed cell death-1 ligand 1 (PD-L1, also known as B7H1 or CD274), and later on of PD-L2 (B7DC or CD273) expression on primary HRS cells with high level of expression of the counter-receptor, programmed death-1 (PD-1) on surrounding T cells is the clinically most relevant finding explaining the immunosuppressive tumor environment (21). Once the link of PD-1/PD-L1-mediated immunosuppression in cHL was established, a potentially effective immunologic strategy for the treatment of cHL was postulated. The hypothesis was supported by laboratory evidence as bulk cHL tumor cells cultured in the presence of anti-PD-L blocking antibodies produced increased amounts of IFN-γ. Furthermore, PD-L blockade was accompanied by the inhibition of SHP-2 phosphorylation known to be a mediator of the PD-1 signaling pathway (22). In turn, depletion or enrichment of T-cell subsets from cHL cell suspension indicated that PD-L blockade restored primarily the function of CD4^+^ T cells of cHL which were already known to be the primary cells of contact surrounding HRS cells in cHL tissue. Following theses data, it was postulated that the antitumor activity of HL-infiltrating T cells was inhibited *via* the PD-1–PD-L signaling pathway, and that this inhibition could be successfully overcome by the use of PD-1/PD-L blocking antibodies.

High PD-L1 expression on HRS cells is caused by a structural amplification on chromosome 9, locus 9p24.1 which leads to a higher expression of PD-L1 and, to a lesser extent, PD-L2 protein (23). The high expression level of PD-L1 is explained by increased JAK2 signaling which further augments PD-1L expression in cell lines with 9p24.1 amplification. Therefore, JAK2 inhibition might be the next rational therapeutic target alone or in combination with PD-1 blockade. This hypothesis is supported by laboratory evidence using commercially available JAK2 inhibitors, demonstrating an excellent correlation between the doses required to inhibit phospho-JAK2 and decreased PD-L1 transcription which reduces the proliferation of cHL cell lines (21). These data may explain the cellular immunodeficiency seen in cHL patients and, moreover, support the further evaluation of PD-1 blockade and JAK2 inhibition, alone and in combination, in patients with cHL characterized by 9p24.1 amplification and its associated targets.

## Immunotherapy of HL

### Identification and Characterization of Potential Target Antigens

The search for target antigens in cHL has resulted in the identification of different molecules with most of them belonging to either the group of lymphocyte (activation) antigens (e.g., CD25, CD30, CD40, and CD80) (24, 25) or molecules of unknown function at the time of discovery (e.g., IRac) (26). Although CD25 antigen and IRac were used in initial studies as potential target molecules, the CD30 antigen is nowadays accepted as probably the best and most reliable marker for the identification of Reed–Sternberg cells. As a consequence, the CD30 antigen is used as target molecule for the treatment of cHL despite its expression in other malignant diseases such as some subtypes of non-HLs, embryonal carcinomas, malignant melanomas, and mesenchymal tumors (24). In addition, CD30 antigen expression is upregulated in some autoimmune diseases as well. The molecular cloning of the extracellular domain of CD30 antigen was done more than 20 years ago (27), and the sequence indicates that it belongs to the nerve growth factor receptor (NGFR) superfamily (28). The CD30 antigen constitutes a 120 kDa type I transmembrane glycoprotein of 578 amino acids and shares common features with TNFR-I, TNFR-II, and NGFR factors, respectively. Biochemical studies of the CD30 molecule provided strong evidence for a signal-transducing role since all CD30 forms are phosphorylated at serine and/or tyrosine residues and its intracellular component possesses kinase activity (29). CD30 signaling activates NF-κB and ERK1/2 and, in some studies, supports the survival of cHL cells (30). Following this observation, it has been speculated for some time that CD30 plays a key role in antiapoptosis and cytokine expression leading to the characteristic histopathological pattern of cHL (31). However, how CD30 contributes to the intracellular signaling network of HRS cells has not been thoroughly investigated. A link between chaperone proteins such as heat shock proteins (HSPs) and CD30 antigen was recently established. Signaling through the CD30 antigen facilitated the phosphorylation of heat shock factor 1 and activated the heat shock promoter element which in turn induced HSP 90 expression (30). The authors could demonstrate that CD30 repression and subsequent inhibition of HSP90 suppressed NF-κB, extracellular signal-regulated kinase, AKT, and STAT pathways in some cHL cell lines. Thus, CD30-mediated induction of HSP90 might serve as a central hub for the integration of intracellular signaling in cHL cells (30).

External CD30 antigen stimulation by soluble recombinant CD30 ligand seems to have a counteractivity on cell survival since the growth of human T-cell lymphoma cell lines *in vitro* is inhibited by apoptosis (32, 33).

### Development of Monoclonal CD30-Specific Antibodies

The CD30 antigen was originally identified on cultured HRS cells using the monoclonal antibody (Mab) Ki-1 (34). Since overexpression of the CD30 antigen has first been described for cHL, CD30 antigen-specific Mabs were originally raised against cell lines from this entity and most Mab-based studies have been performed in this entity (35). The first-generation CD30 antigen-specific Mabs raised in the 1980s (e.g., Ki-1, BerH2, and HRS1–4) (36) had no effect on cultured HRS cell lines and showed no signs of activity in early clinical trials. However, they demonstrated rapid cellular internalization after binding to the CD30 antigen. Their potential therapeutic role was believed to be outside of the so-called unconjugated antibody field and more in the area of delivery vehicle for cytostatic drugs (37), plant toxins (38), or a number of chemically linked immunotoxins (ITs) with some of them being developed and evaluated for clinical application (39–41) as delineated in the following paragraphs. The clinical development of these antibodies was supported by biodistribution studies in cHL patients (42), performed in the early 1990s. In these trials, specific tumor targeting with positive imaging could be confirmed for the first-generation CD30-specific antibody HRS-3. As a consequence, this antibody was used by our group as the tumor-targeting backbone for the development of different constructs.

However, at the same time, other groups had developed second-generation CD30-specific antibodies recognizing different CD30-epitopes and could demonstrate *in vitro* activity by growth inhibition of cultured cell lines and, in some instances, direct *in vivo* efficacy by reduced growth of tumor xenografts in SCID mouse models. This effect was not observed with first-generation CD30-specific antibodies such as BerH2. The precise mechanism underlying this inhibition remained unknown, and the authors speculated that the second-generation CD30-specific Mabs were directed against the CD30-ligand-binding site and, therefore, might directly affect antigen–ligand interaction resulting in impaired cell growth (43). However, conflicting data were published subsequently since some second-generation Mabs such as 5F11 activated the NF-κB pathway and the antiapoptotic protein cellular FLICE (Fas-associating protein with death domain-like interleukin-1β-converting enzyme) inhibitory protein (c-flip) causing apoptosis resistance and, thus, limiting the potential clinical use of 5F11. To overcome this resistance, 5F11 had to be combined with proteasome inhibitors such as bortezomib and this combination demonstrated a synergistic cytotoxic effect *in vitro* and in a human cHL xenograft model provided that 5F11 preceded bortezomib treatment (44).

Nevertheless, the data on the second-generation CD30-specific Mabs sparked renewed interest in the clinical use of unconjugated CD30-specific antibodies and resulted in multiple clinical trials treating relapsed and refractory patients with CD30^+^ lymphomas with CD30-specific Mabs. These trials have evaluated primarily chimeric or even fully human antibodies such as cAC10 (SGN-30) or 5F11 (MDX-60), respectively.

#### Clinical Development of SGN-30

The chimeric CD30-specific cAC10 Mab (SGN-30) was tested in a pivotal phase II study for efficacy after having passed classical dose-escalation phase I protocols without dose-limiting toxicity (45). In this trial, the objective response rate (ORR) was 17%, reaching 25% for the 28 anaplastic large cell lymphoma (ALCL) patients who had received at least one full course of SGN-30. Although a meaningful number of cHL patients (29%) achieved a stable disease (SD), no objective responses (ORs) to SGN-30 were observed. The dose of SGN-30 was increased to 12 mg/kg on weekly administration following an interim analysis of the safety data. Again, with a limited number of patients in each group, no relationship between antitumor activity or safety and dose level in either cHL or ALCL patients was seen, respectively. The authors speculated that the modest antitumor activity of SGN-30 in ALCL with almost no effect in cHL patients may reflect the limited number of CD30^+^ HRS cells accessible in cHL tumors which are significantly less per tumor volume when compared with the homogeneous expression of CD30^+^ lymphoma cells in ALCL tumors. The collective results of this phase II study demonstrated that SGN-30 administered to patients with relapsed or refractory cHL and systemic ALCL was well tolerated since only three of 79 patients (4%) presented with hypersensitivity or allergic reactions, respectively. Therefore, the acceptable safety profile of SGN-30 and the modest observed antitumor activity supported the use of this agent as antibody drug conjugate.

#### Clinical Development of MDX-060

The fully human CD30-specific Mab MDX-060 (5F11, iratumumab) was tested in a similar setting, and doses up to 15 mg/kg were administered without dose-limiting toxicity (46). Although clinical responses were seen in both patient subgroups (cHL and ALCL) at most dose levels ≥1 mg/kg, the ORR was only 8%. Two (28%) of the seven ALCL patients had a response, compared with 6% of patients (4 of 63) with cHL. Disease stabilization was observed in 35% of patients, similar to one seen in SGN-30 trials. Four of six responding patients had received corticosteroids while on study making it difficult to attribute the efficacy observed to the antibody alone. Once again, the results of this study indicate that MDX-060 administration to patients with relapsed or refractory CD30 expressing lymphomas was well tolerated. However, due to its limited clinical value, the future development of MDX-060 was abandoned.

### Antibody-Based Radio-Immunotherapy (RIT)

As described earlier, biodistribution trials using radiolabeled first-generation CD30-specific HRS-3 Mab had demonstrated favorable uptake in cHL tissue, and the development of radioimmunoconjugates (RICs) was a logical next step (47). The rational for RIC is supported by the well-known clinical experience of high sensitivity of cHL to ionizing radiation. In a pivotal phase I/II clinical trial, 22 patients with biopsy proven CD30^+^ cHL were included. Most patients presented with advanced-stage disease (19 of 22 patients) and were heavily pretreated with a median of four different prior chemotherapy regimes (range, 2–6) including high-dose chemotherapy (HDT) and autologous stem-cell transplantation (ASCT) in 16 of 22 patients. Iodine-131 was selected as radioisotope since it is readily available and allows for dosimetry after trace doses of the RIC (day 1) followed by a therapeutic dose on day 8. As reported by the authors, results were disappointing since visualization of tumor masses was seen only in a minority of patients (23%). Moreover, measurable tumor responses were limited and included one CR, five PRs, and three MRs, which lasted for a median of 4 months. In general, acute toxicity was mild with transient fatigue in 86% and nausea in 23% of patients. The most relevant toxicity in this heavily pretreated patient population was severe myelosuppression as seen in 33% of all patients. Against expectations, there was neither a correlation between toxicity observed and number of prior treatment lines, administered whole-body dose or laboratory values prior to treatment preventing the definition of a most optimal dose for further studies. As a consequence, RIT in HL has not been further explored.

### Antibody-Based ITs

Delivery of highly cytotoxic reagents by an antibody construct at the tumor site has been an attractive concept for quite some time and was supported by the availability of first-generation CD30-specific antibodies with favorable tumor-targeting properties in cHL patients. As part of early laboratory studies, Engert and colleagues had analyzed five CD30-specific Mabs antibodies and two derived Fab′ fragments linked to deglycosylated ricin A chain (dgA) for their potential to act as ITs for the treatment of cHL (48). Once again, the first-generation CD30-specific Mab HRS-3 turned out to be the most optimal candidate based on its high tumor antigen affinity (*K*_d_ 15 nM) and high activity as measured by inhibition of protein synthesis of L540 cHL cells by 50% [0.9 × 10(−10) M]. HRS-3.dgA was chosen as the preferred IT as it was only 15 times less toxic than the toxin ricin itself (49). HRS-3.dgA was later replaced by an even more potent IT (Ki-4.dgA) which was five times more potent *in vitro* and displayed high efficacy in the treatment of disseminated human cHL when studied in SCID mice xenografts (50). Thus, Ki-4.dgA was selected for a clinical phase I trial in 16 patients with refractory CD30^+^ lymphoma (25). The maximal tolerated dose (MTD) was lower than expected and established at 5 mg/m^2^. The authors speculated that binding of the IT to sCD30 and prolonged persistence of sCD30/IT complexes in the blood might have been a factor contributing to higher toxicity. Dose-limiting toxicities were hypoalbuminemia, weight gain, tachycardia, hypotension, dyspnea, weakness, and fatigue. Additional side effects included myalgia, nausea, and vomiting. Response rates were moderate with one PR, one MR, two SD, which is similar to other studies using, for example, CD25.dgA constructs in a similar patient population. More importantly, 7 of 17 patients (one patient with ALCL) developed human-anti-Ricin-A antibodies (HARA) and in 1 of 17 patients human-anti-mouse-antibodies (HAMA) against the antibody backbone were detected. Both, HAMA and HARA might limit the number of applicable IT courses and prevent further treatment cycles. At that time, it was clear that the future development of antibody-based ITs for cHL treatment needed improvement in three major areas:

1. Less immunogenic antibodies (or their fragments) of either chimeric or human/humanized nature
2. Less immunogenic but still very potent toxin compound
3. Optimal conjugation (the so-called linker) between the antibody and toxin moiety. The linker should be stable enough to prevent unwanted toxin release from the antibody in blood circulation but still allow for rapid toxin release once the antibody construct had been internalized by the HRS cells.

#### Brentuximab Vedotin (BV)

It took a long time and huge effort to achieve the three aforementioned goals until a clinically successful IT construct was established: the antitubulin agent monomethyl auristatin E (MMAE) was attached to the already mentioned CD30-specific Mab cAC10 by an enzyme-cleavable dipeptide linker generating the antibody–drug conjugate BV (SGN-35) (51). The antibody–drug conjugate is rapidly internalized after binding to the CD30 antigen and transported to lysosomes, where the peptide linker is selectively cleaved. The toxin MMAE is then released into the cell, binds to tubulin, and prompts cell cycle arrest between the Gap 2 phase and mitosis (G2/M) leading to cell apoptosis (51).

##### BV Treatment of Relapsed and/or Refractory HL Patients

After successful preclinical tests demonstrating high and selective activity against CD30^+^ tumor-cell lines *in vitro* and *in vivo* xenograft models, a phase I, open-label, dose-escalation trial was initiated (52). Of the 45 patients treated, 42 had cHL, 2 systemic ALCL, and 1 CD30^+^ angioimmunoblastic T-cell lymphoma. As characteristic for cHL patients with relapsed and/or refractory disease, patients were of young age (36 years; range, 20–87) and had undergone multiple lines of prior treatment (median of three previous chemotherapy regimens with a range from 1 to 7). In addition, 33 patients (73%) had undergone previous HDT followed by ASCT. In contrast to previous IT trials in cHL, tumor responses were observed in the majority of patients treated with BV with tumor regression in 86% of patients. Tumor-related symptoms ameliorated in 81% of those in whom such symptoms were present at the time of treatment initiation. Seventeen patients achieved an OR including 11 CRs. Six of 12 patients (50%) receiving the maximum tolerated dose had an OR suggesting a potential relationship between administered dose and efficacy. Remissions were durable in this patient population who had relapsed or refractory disease at study entry. The median duration of response (DOR) was at least 9.7 months.

Side effects included mainly grade one or two fatigue, pyrexia, diarrhea, nausea, neutropenia (with one grade three event), and peripheral neuropathy at the MTD. Standard supportive care controlled most adverse events that were typically of grade 1 or 2. Clinically relevant is the cumulative, dose-related grade 1 or 2 peripheral neuropathy caused by the MMAE toxin as potent antitubulin agent. This toxicity is known to be a class effect of microtubule inhibitors (53).

The phase I trial was followed by a multicenter phase II trial of BV monotherapy in a total of 102 patients with relapsed or refractory cHL (54). Patients were treated with BV 1.8 mg/kg by intravenous infusion every 3 weeks. Patients received a maximum of 16 cycles in the absence of disease progression or prohibitive toxicity. Tumor reductions were common and seen in 94% of all patients treated. As confirmed by independent review, 75% of patients achieved an OR with 34% obtaining a CR. The median progression-free survival (PFS) was 5.6 months, and the median DOR for CR patients was 20.5 months. Thirty-one patients were still alive and free of documented progressive disease after a median observation time of more than 1.5 years. The cohort of patients included in the trial was particularly refractory to prior treatments as evidenced by the fact that 71% of patients did not achieve a CR or had experienced a relapse within 3 months following frontline therapy. Furthermore, these patients had a poor prognosis because the median time to relapse after HDT + ASCT was only 6.7 months. In this context, the OR rates and durable CRs are quite impressive for a single-agent therapy considering the failure of prior combination chemotherapies including HDT plus ASCT. It is a common observation that each successive treatment delivered to a patient with relapsed lymphoma results in diminishing remission times, usually cut by half with every additional line of treatment. Therefore, it was encouraging to see that the PFS achieved with BV was significantly longer than the one achieved with the most recent prior therapy in the subset of patients who had received a systemic therapy after HDT plus ASCT.

In this study, BV was administered for a maximum of 16 cycles; the actual median and mean durations of treatment were 9 and 10 cycles, respectively. Although the majority of responses occurred early in the course of treatment, one CR was initially documented after approximately 1 year of therapy. Eighteen patients received all 16 treatment cycles. One has to admit that the optimal treatment duration is unknown and not answered by the present trial. However, peripheral neuropathy is usually the most frequent dose-limiting side effect making it in general quite unlikely that treatment is continued even beyond 16 cycles. Peripheral neuropathy typically develops after prolonged BV exposure with a median onset of grade 2 at 27.3 weeks (eight to nine treatment cycles). Peripheral neuropathy was largely reversible since dose reductions or even cessation of treatment was done promptly (54). Twenty-three percent of all patients entering the trial had already existing peripheral neuropathy as they had been exposed to neurotoxic drugs in their previous lines of treatment.

In summary, these two pivotal phase I/II trials were a major achievement in the process of establishing IT for the treatment of relapsed/refractory cHL since they had demonstrated and confirmed the safety and efficacy for the chosen compound. Moreover, they had a significant impact beyond cHL treatment since they laid the ground for the development of additional ITs in other tumor entities. From this trial onward, efficacy and to some extent dosing for BV was established and drug development to find the most optimal setting for clinical use of BV was started.

##### BV Consolidation in cHL Patients After High-Dose Chemotherapy and Autologous Stem Cell Support

Since BV is effective in detecting and eliminating CD30^+^ cells, concepts for an early use of BV at the stage of minimal residual disease (MRD) were developed. One approach of using BV as consolidation treatment in cHL patients at high risk of relapse after HDT plus ASCT was tested in the AETHERA trial (55). As generally seen in aggressive lymphoma, relapse or progression after front-line or even second-line treatment including HDT plus ASCT happens generally early. After HDT plus ASCT, 71% of progression events are observed within 1 year of transplant, and 90% will happen within 2 years. Therefore, patients passing the 2 years’ time period without relapse have usually a high change of being cured (55).

The results from the AETHERA trial demonstrated that consolidative treatment with BV compared with placebo provided a statistically and clinically significant improvement in PFS. By independent review, the estimated proportion of patients who were alive, and progression free was 63% with BV vs. 51% with placebo at 24 months. By investigator assessment, the estimated 24 months PFS data were very similar with 65 and 45%, respectively. Overlooking 108 patient-years of follow-up, only four PFS events were detected after the 24 months assessment period. In addition to the sustained clinical benefit of BV consolidation, more patients needed subsequent antitumor therapies in the placebo group than in the BV group, including nearly twice as many allogeneic stem-cell transplantations (55). These data suggest that the early use of BV after HDT plus ASCT can control MRD and might be beneficial in the long term since it might spare subsequent, sometimes quite intensive treatments.

The PFS benefit for BV treated patients was seen across all prespecified subgroups, including primary refractory patients and patients who had relapsed less than 12 months after frontline therapy (55). These two patient cohorts are generally seen as the ones with the worst overall prognosis and low chances of being cured by subsequent therapies. Compared with historical survival data for high-risk patients with Hodgkin’s lymphoma undergoing HDT plus ASCT, the 3-year OS rate in this study was remarkable exceeding 80% and underlined the clinical benefit of BV treatment as consolidation therapy and as rescue therapy, respectively. However, the study did not conclusively answer the question if BV should be used in all cHL patients after HDT plus ASCT to control MRD or if it should be spared for relapsing patients as salvage therapy. It is tempting to speculate that early BV consolidation will result in a reduced number of progression events and more patients might be cured with consolidation therapy. We think it is fair to agree with the authors that reduced numbers of patients will need subsequent toxic therapy for active disease including allogeneic stem-cell transplantation.

##### BV First-Line Treatment in Patients With Advanced cHL

Very recently, data from the randomized phase III ECHELON-1 trial were presented at the ASH meeting 2017 and published (56). The trial compared BV as part of a combination chemotherapy regimen (BV plus doxorubicin, vinblastine, and dacarbazine; A + AVD) against the standard ABVD (doxorubicin, bleomycin, vinblastine, and dacarbazine) chemotherapy in cHL patients with advanced stage (III or IV) disease and had not been previously treated with systemic chemotherapy or radiotherapy. The ECHELON-1 trial met its primary endpoint and showed a statistically significant improvement in a so-called modified progression-free survival (mPFS) endpoint. The 2-year rate of mPFS was 82% in the A + AVD group [95% confidence interval (CI), 78.7–85.0] compared with 77% (95% CI, 73.7–80.4) in the ABVD group. The hazard ratio for progression, death, or modified progression was 0.77 (95% CI, 0.60–0.98; *P* = 0.03).

From today’s perspective, it is obvious that BV will soon be incorporated into standard first-line chemotherapy regimen of cHL. However, it is not yet clear if the combination partner will be AVD as in the present trial or other regimens such as BEACOPP variants. cHL patients with advanced-stage disease in need of a frontline chemotherapy are currently studied with BV in the BrECADD HD21 regimen as part of the German HL study group (NCT02661503).

### Bispecific Antibodies

Bispecific antibodies (BiMabs) are typically designed to bind simultaneously to the tumor cells and to a trigger receptor on immune effector cells (57), for example the FcγRIII (CD16) on natural killer (NK) cells or CD3 receptor on T cells, respectively. For the treatment of cHL, we had established NK- and T cell stimulating BiMabs and demonstrated their efficacy *in vitro* and *in vivo* using different xenograft mouse models (58–62).

The CD30/CD16 BiMab HRS-3/A9 was chosen for clinical development and produced under GMP conditions confirming the general applicability of this approach. Fifteen patients with refractory cHL were treated in a phase I/II trial with the BiMab HRS-3/A9 (62). The BiMab was administered four times every 3–4 days, starting with 1 mg/m^2^. The treatment was well tolerated, and the MTD was not reached at the highest dose administered (64 mg/m^2^) because of limited amounts of available antibody. BiMab HRS-3/A9 induced no DLT but only short-lasting mild to moderate side effects occurred in a minority of patients. Nine patients (60%) developed an HAMA response as determined by ELISA 4 weeks after treatment. At that time, a second treatment cycle was intended in four patients, of whom three were HAMA-positive. As defined in the protocol, HAMA-positive patients were challenged with intracutaneous BiMab HRS-3/A9 application, and one patient who had presented with an allergic skin rash after the first BiMab treatment cycle developed a marked skin reaction with erythema and induration and, therefore, was excluded from further treatment. The remaining three patients with negative skin tests received a second BiMab infusion at the dose level that they had tolerated during the first treatment cycle. In all retreated patients (including one who had been HAMA negative), moderate systemic reactions such as shivering, hypotension, low back pain, and chest tightness occurred despite pretreatment with anti-histamins and prednisone leading to the termination of treatment after one to three additional BiMab infusions. In total, the ORR was 33% with one CR and one PR (lasting 6 and 3 months, respectively) as well as three MRs lasting for 1–15 months. Our results emphasized the necessity to reduce the immunogenicity of the murine BiMab construct but at the same time encouraged us to develop this novel immunotherapeutic approach further.

Based on these data, the objectives of a second phase I trial were the evaluation of a modified BiMab application schedule with a prolonged infusion time designed to provide a higher antitumor efficacy and/or a better tolerance of retreatment attempts, respectively (63). Finally, because patients with advanced cHL generally show a severe qualitative and quantitative immunosuppression as outline before, and because the number and degree of activation of NK cells are crucial for this immunotherapeutic approach, the influence of additional cytokine co-stimulation was evaluated. Therefore, patients achieving an SD after the first course of BiMab treatment were scheduled for a second BiMab course adding concomitant IL-2 and GM-CSF as immune stimulation. Infusions were given either as continuous infusion over 24 h on four consecutive days or as a standard 1 h infusion, respectively. In summary, patients had received a total of 27 BiMab courses, including six courses with co-administration of cytokines. At re-evaluation after the first treatment cycle, two PRs and six cases of SD were observed, whereas treatment was stopped in the remaining eight patients because of progressive disease. The cumulative ORR after BiMab treatment was 25%, with one CR lasting for 6 months and three PRs lasting for 3, 5, and 9 months, respectively. Continuous infusion seemed to be the superior application regimens with three of the four OR in this treatment arm. In addition, four disease stabilizations (after documented preceding PD) lasting for 3 to more than 6 months (the latter in a patient finally undergoing allogeneic bone marrow transplantation with a fatal outcome) were observed. Toxicity proved again to be very low, with transient mild to moderate fever as the major side effect occurring in about one-third of the patients. A BiMab-directed HAMA response occurred in 37.5% of our patients within 4 weeks after treatment. This is in line with incidences between 40 and 84% observed in other clinical trials with murine BiMabs (64, 65) and our previous study (46%) (62). As a result of the two studies, we postulated that redirecting NK cells by CD16-specific BiMab was a promising approach but needed significant technical improvement to obtain an antibody construct with lower or ideally missing immunogenicity, high activity, and good productivity under GMP conditions.

These prerequisites might be fulfilled by a bispecific, tetravalent chimeric antibody construct (TandAb) called AFM13 (66, 67). This antibody construct specifically recruits NK cells since it only recognizes the CD16A isoform on NK cells and does not cross-react with granulocytes. TandAbs have two binding sites for each antigen, but no Fc domains and can be produced at large scale in mammalian cells. Preclinical data have demonstrated a specific and efficient antitumor activity against CD30^+^ target cells by the engagement of NK cells. After passing extensive preclinical tests, a phase I study with AFM13 in heavily pretreated cHL patients who had received all standard therapies was initiated (66). Since no appropriate *in vivo* model for safety and efficacy was available, AFM13 dosing started at very low levels and was then gradually escalated by 700-fold. Treatment with AFM13 was well tolerated at all dose levels and the MTD not reached. Side effects were generally mild with moderate AEs. A PR (11.5%) was seen in 3 of 26 evaluable patients, and 13 patients achieved disease stabilization (50%) leading to an overall disease control rate (DCR) of 61.5%. A dose–response dependency could be seen since 13 patients treated with AFM13 doses of ≥1.5 mg/kg had an ORR of 23% and the DCR was 77%, respectively. Important regarding the most optimal scheduling of IT and BiMab therapies was the observation that AFM13 was also active in BV-refractory patients. As expected, AFM13 treatment resulted in a significant NK-cell activation and a decrease of sCD30 in the peripheral blood. As stated by the authors, AFM13 treatment was safe and demonstrated reasonable activity in this heavily pretreated patient cohort. The results obtained so far warrant further development of this construct at earlier stages of disease or even in combination with other immunotherapeutic approaches as described below.

### Check-Point Blockade Inhibiting Antibodies

Check-point blockade inhibiting antibodies have changed the treatment paradigm of many solid organ cancers and revived our belief that the immune system can control and even eradicate cancer cells (68). CTLA-4 and/or PD-1/PD-L1 blocking antibodies have established themselves in the first-line treatment of so far difficult to treat cancers such as advanced-stage melanoma or lung cancer, respectively. cHL was not the prime target for check-point blockade inhibiting antibodies, mainly because effective treatment options at diagnosis and relapsed were available. That changed when the abovementioned association between chromosome 9p24.1 amplification and enhanced expression of the PD-1 ligands on HRS cells was detected. This created a rational link between the immunosuppressive state observed in cHL tissue and a potential therapeutic option by using Mabs against PD-1/PD-ligands blocking the interaction of these molecules and unleashing the immune response against HRS cells. Nowadays, cHL is known as the disease with the highest response rates toward check-point blockade inhibitor treatment and time will tell, if cHL patients at relapsed can be cured by this treatment (69). A first landmark study using the PD-1 blocking antibody nivolumab was published in 2015 (70). In this study, 23 patients with relapsed or refractory cHL had been enrolled. The median age of patients at stud entry was 35 years (range 20–54 years), and 17 patients (74%) had an ECOG performance-status score of 1e. Since first- and second-line treatment in cHL is well established, all patients entering the trial had been extensively pretreated with 87% having received three or more previous treatment regimens. Moreover, 78% of the patients had received BV, and the same number of patients had undergone HDT plus ASCT. The DCR was 87% (95% CI, 66–97), with 4 patients (17%) reaching a CR, 16 patients (70%) a PR, and 3 patients (13%) with disease stabilization. The response rate was unchanged when only patients (*n* = 15) with disease recurrence after HDT plus ASCT and BV treatment were analyzed. All three patients who had not received prior HDT + ASCT but BV treatment achieved a PR leading to a response rate of 100% (95% CI, 29–100). Besides the high ORR, DOR was impressive as well with a PFS of 86% at 24 weeks. Adverse events were similar to the known toxicity profile as seen in solid organ tumors and mainly of grade 1 or 2. The high efficacy of PD-1 blockade in cHL is not restricted to nivolumab alone but has been confirmed for the alternative PD-1 blocking antibody (pembrolizumab) and was shown by a large phase II trial in 210 patients with relapsed and/or refractory cHL (71). The study focused more closely on responses and DOR in three different patient subpopulations as defined by relapse after HDT plus ASCT and subsequent BV treatment (cohort 1); salvage chemotherapy and BV but ineligible for ASCT because of chemoresistant disease (cohort 2); and HDT plus ASCT but without BV after transplantation (cohort 3). Cohort 1 is the more classical group of patients who receive all available treatments but relapse over time. Patients received a flat dose of pembrolizumab 200 mg once every 3 weeks. Overall, the ORR was slightly lower than in the nivolumab trial with 73%. However, the patient composition in the pembrolizumab trial was more unfavorable and more patients with refractory (*n* = 170) or even primary refractory disease (*n* = 73) were included. From a clinical perspective, patients with primary refractory disease need special attention since they can hardly be rescued by any subsequent treatment. It was encouraging to see that the ORR was 79.5% (95% CI, 68.4–88.0) in this patient subgroup. This was even higher than the ORR in patients from cohort (64.2%; 95% CI, 52.8–74.6). With still short follow-up, median OS continued at time of analysis and was not reached. The authors reported a 9 month OS and PFS rates of 97.5 and 63.4%, respectively (71). So far, none of the PD-1 antibody trials could reveal a robust predictive biomarker identifying either those patients who might have the greatest benefit or those where PD-1 blockade is not sufficient and further support by other approaches is needed. One retrospective analysis addressed this issue for cHL patients after nivolumab therapy and identified MHC class II expression beside PD-L1 as potential marker (72). Positive MHC class II expression on HRS cells was predictive for prolonged PFS in patients who had received the PD-1-blocking antibody >12 months after ASCT. These data would argue for an important role of CD4^+^ T cell activation by PD-1 blockade as mechanism of action in cHL and, potentially, other PD-L1-positive tumors.

In summary, the success of PD-1 blockade by Mabs has spurred the search for other lymphoma entities with constitutive PD-L overexpression and will change the treatment paradigm in cHL, certainly for patients with primary refractory disease and those with multiple relapse. PD-1 blockade can be combined with other established or novel therapies, and Table 1 summarizes the ongoing studies of PD-1 blocking antibodies in cHL treatment as listed on http://clinicaltrials.gov. These trials cover the full spectrum of combinations with first/second-line chemotherapy, ITs (BV), bispecific antibodies (AFM13), immunomodulators, radiotherapy, and novel TKIs. Hopefully, new combinational therapies will secure the high cure rates seen in cHL with less long-term toxicity and, eventually, replace classical chemotherapy.

**Table 1:** Ongoing trials in classical Hodgkin lymphoma with programmed death-1 blocking antibodies as listed on http://clinicaltrials.gov.

	Title	Study drug(s)	NCT no.
1	A Study of Safety and Efficacy of Nivolumab and Bendamustine (NB) in Patients With Relapsed/Refractory Hodgkin’s Lymphoma	Nivolumab Bendamustine	03343652
2	Treatment With Nivolumab at the Fixed Dose 40 mg (Nivo40) in Patients With Relapsed/Refractory Hodgkins Lymphoma	Nivolumab	03343665
3	A Study of Brentuximab Vedotin Combined With Nivolumab for Relapsed or Refractory Hodgkin Lymphoma	Nivolumab Brentuximab vedotin (BV)	02572167
4	Ibrutinib and Nivolumab in Treating Patients With Relapsed or Refractory Classical Hodgkin Lymphoma	Nivolumab Ibrutinib	02940301
5	Study of Nivolumab in Patients With Classical Hodgkin’s Lymphoma (Registrational)	Nivolumab Doxorubicin Vinblastine Dacarbazine	02181738
6	Nivolumab and AVD in Early-stage Unfavorable Classical Hodgkin Lymphoma	Nivolumab Adriamycin Vinblastine Dacarbazine	03004833
7	A(B)VD Followed by Nivolumab as Frontline Therapy for Higher Risk Patients With Classical Hodgkin Lymphoma	Nivolumab Doxorubicin Bleomycin Vinblastine Dacarbazine	03033914
8	A Study of Nivolumab Plus Brentuximab Vedotin in Patients Between 5 and 30 Years Old, With Hodgkin’s Lymphoma (cHL), Relapsed or Refractory From First Line Treatment	Nivolumab BV Bendamustine	02927769
9	A Study of Nivolumab Plus Brentuximab Vedotin Versus Brentuximab Vedotin Alone in Patients With Advanced Stage Classical Hodgkin Lymphoma, Who Are Relapsed/Refractory or Who Are Not Eligible for Autologous Stem Cell Transplant	Nivolumab BV	03138499
10	Brentuximab Vedotin and Nivolumab With or Without Ipilimumab in Treating Patients With Relapsed or Refractory Hodgkin Lymphoma	Nivolumab BV Ipilimumab	01896999
11	Nivolumab, Ifosfamide, Carboplatin, and Etoposide as Second-Line Therapy in Treating Patients With Refractory or Relapsed Hodgkin Lymphoma	Nivolumab Carboplatin Etoposide Ifosfamide	03016871
12	Nivolumab and Brentuximab Vedotin After Stem Cell Transplant in Treating Patients With Relapsed or Refractory High-Risk Classical Hodgkin Lymphoma	Nivolumab BV	03057795
13	Nivolumab and Brentuximab Vedotin in Treating Older Patients With Untreated Hodgkin Lymphoma	Nivolumab BV	02758717
14	Nivolumab and Ipilimumab in Treating Patients With HIV Associated Relapsed or Refractory Classical Hodgkin Lymphoma or Solid Tumors That Are Metastatic or Cannot Be Removed by Surgery	Nivolumab Ipilimumab	02408861
15	Safety of Allogeneic Hematopoietic Cell Transplantation (HCT) For Patients With Classical Hodgkin Lymphoma (CHL) Treated With Nivolumab	Non-interventional	03200977
16	Study to Assess the Safety of Nivolumab in the Treatment of Metastatic Melanoma, Lung Cancer, Renal Cancer, Squamous Cell Carcinoma of the Head and Neck, and Chronic Hodgkin Lymphoma in Adults in Mexico	Non-interventional	03161613
17	A Study of Brentuximab Vedotin in Adults Age 60 and Above With Newly Diagnosed Hodgkin Lymphoma (HL)	Nivolumab BV Bendamustine	01716806
18	Brentuximab Vedotin With or Without Nivolumab in Treating Patients With Relapsed or Refractory CD30^+^ Lymphoma	Nivolumab BV	01703949
19	Nivolumab With Epstein Barr Virus Specific T Cells (EB-VSTS), Relapsed/Refractory EBV Positive Lymphoma (PREVALE)	Nivolumab EB-VST cells	02973113
20	Nivolumab With or Without Ipilimumab in Treating Younger Patients With Recurrent or Refractory Solid Tumors or Sarcomas	Nivolumab Ipilimumab	02304458
21	Ipilimumab or Nivolumab in Treating Patients With Relapsed Hematologic Malignancies After Donor Stem Cell Transplant	Nivolumab Ipilimumab	01822509
22	Trigriluzole With Nivolumab and Pembrolizumab in Treating Patients With Metastatic or Unresectable Solid Malignancies or Lymphoma	Nivolumab Pembrolizumab Trigriluzole	03229278
23	Pembrolizumab and Involved Site Radiation Therapy for Early Stage Relapsed or Primary Refractory Hodgkin Lymphoma	Pembrolizumab Involved site Radiation therapy	03179917
24	Study of the Combination of AFM13 and Pembrolizumab in Patients With Relapsed or Refractory Classical Hodgkin Lymphoma	Pembrolizumab AFM13	02665650
25	Study of Pembrolizumab (MK-3475) vs. Brentuximab Vedotin in Participants With Relapsed or Refractory Classical Hodgkin Lymphoma (MK-3475-204/KEYNOTE-204)	Pembrolizumab BV	02684292
26	Study of Pembrolizumab (MK-3475) in Participants With Relapsed or Refractory Classical Hodgkin Lymphoma (MK-3475-087/KEYNOTE-087)	Pembrolizumab	02453594
27	Safety & Efficacy Study of Combination of Pembrolizumab and Lenalidomide, in Patients With Relapsed Non-Hodgkin and Hodgkin Lymphoma	Pembrolizumab Lenalidomide	02875067
28	Pembrolizumab After ASCT for Hodgkin Lymphoma, DLBCL and T-NHL	Pembrolizumab	02362997
29	PET-Directed Therapy With Pembrolizumab and Combination Chemotherapy in Treating Patients With Previously Untreated Classical Hodgkin Lymphoma	Pembrolizumab Dacarbazine Doxorubicin Vinblastine	03226249
30	Pembrolizumab and Combination Chemotherapy in Treating Patients With Relapsed or Refractory Hodgkin Lymphoma	Pembrolizumab Carboplatin Etoposide Ifosfamide	03077828
31	Pembrolizumab and Vorinostat in Treating Patients With Relapsed or Refractory Diffuse Large B-Cell Lymphoma, Follicular Lymphoma, or Hodgkin Lymphoma	Pembrolizumab Vorinostat	03150329
32	Pilot Study of Pembrolizumab Treatment for Disease Relapse After Allogeneic Stem Cell Transplantation	Pembrolizumab	02981914
33	A Study of Pembrolizumab (MK-3475) in Pediatric Participants With an Advanced Solid Tumor or Lymphoma (MK-3475-051/KEYNOTE-051)	Pembrolizumab	02332668
34	Pembrolizumab in Treating Patients With HIV and Relapsed, Refractory, or Disseminated Malignant Neoplasms	Pembrolizumab	02595866
35	ACP-196 (Acalabrutinib) in Combination With Pembrolizumab, for Treatment of Hematologic Malignancies	Pembrolizumab Acalabrutinib	02362035
36	A Trial of Pembrolizumab (MK-3475) in Participants With Blood Cancers (MK-3475-013/KEYNOTE-013)	Pembrolizumab Lenalidomide	01953692
37	Pembrolizumab and Ibrutinib in Treating Patients With Relapsed or Refractory Non-Hodgkin Lymphoma	Pembrolizumab Ibrutinib	02950220
38	Safety Study of SEA-CD40 in Cancer Patients	Pembrolizumab SEA-CD40	02376699

## Cellular Therapies: CD30-Specific Chimeric Antigen Receptors (CAR) T Cell Constructs

Today, it is impossible to conclude a review on new immunotherapeutic approaches in malignant hematological diseases without addressing new cellular therapies. The reprogramming of (autologous) T cells with CAR has received FDA approval for the treatment of relapsed juvenile B-ALL and DLBCL and is currently evaluated in additional diseases including cHL (73, 74). Again, the CD30 antigen is the most promising target antigen for CAR T cell approaches in cHL, and preliminary *in vitro* (75) and *in vivo* (76) experiments have revealed promising data.

Recently, data from a phase I dose-finding trial with nine patients suffering from relapsed/refractory EBV-negative cHL and ALCL using autologous CD30scFv-CAR T cells were published (77). Clinical responses were seen in three of nine patients (two CR, one continued CR) with additional three patients achieving an SD. CAR T cells were well tolerated, and the highest degree of expansion of circulating CAR T cells was detected within the first week after infusion in a dose-dependent manner achieving the highest values at the third dose level. Seven patients received a second infusion of CAR T cells with one patient receiving a total of four infusions. However, this resulted only in a modest expansion of CD30-specific CAR T cells in the peripheral blood. One explanation for the modest expansion and low persistence of CAR T cells could be the omission of a lymphodepleting chemotherapy before CAR T cell infusion. This procedure is routinely used in CAR T cell studies for leukemia or lymphoma treatment and has increased CAR T cell persistence quite substantially (74). Since single cases have shown a synergistic effect of PD-1 blockade and CAR T cells in PD-L^+^ lymphoma patients, the combination of CD30-specific CAR T cells and PD-1 blocking antibodies in cHL patients is intriguing and might reveal synergistic activity.

## HL: A Rare Disease with Unique Features

Classical Hodgkin lymphoma is a rare but unique disease and research in cHL has been the pioneer for many medical breakthroughs: cHL was first described in 1832 as deadly disease until the mid-twentieth century. Then, radiotherapy and later on polychemotherapy changed the course of the disease dramatically with nowadays the majority of patients being cured. ITs such as BV were first established for the treatment of relapsed cHL and paved the way for ITs to be accepted as treatment modality in various tumors. Most recently, the immunosuppressive nature of HRS cells was identified to be caused by the constitutive high expression of PD-L, and the rational use of PD-1/PD-L1 blocking antibodies has shown the highest activity in all tumor entities studied so far. With a plethora of therapeutic options, the challenge for future trials will be to design novel study protocols that maintain the high efficacy of their predecessors and abolish the short- and long-term side effects of contemporary standard therapies. Especially, fertility preservation, cardiac and pulmonary toxicity, and lastly neuropathy are issues that need to be addressed. In this regard, collaboration of international study groups will be key to advance treatment in cHL and support clinicians to choose wisely for their patients. Such an effort to combine classical treatment procedures with all the abovementioned immunotherapeutic approaches shall lead to a cure of cHL in all patients with minimal side effects. That would be the next milestone to focus on in the history of cHL.

## Author Contributions

CR and FS have contributed equally to this work.

## Conflict of Interest Statement

The authors declare that the research was conducted in the absence of any commercial or financial relationships that could be construed as a potential conflict of interest.
